# A Novel De Novo Mutation of the *TITF1/NKX2-1* Gene Causing Ataxia, Benign Hereditary Chorea, Hypothyroidism and a Pituitary Mass in a UK Family and Review of the Literature

**DOI:** 10.1007/s12311-014-0570-7

**Published:** 2014-06-15

**Authors:** Liana Veneziano, Michael H. Parkinson, Elide Mantuano, Marina Frontali, Kailash P. Bhatia, Paola Giunti

**Affiliations:** 1 Institute of Translational Pharmacology, National Research Council, Via Fosso del Cavaliere 100, 00133 Rome, Italy; 2 Department of Molecular Neuroscience, UCL Institute of Neurology, Room 712, Queen Square, London, WC1N 3BG UK; 3 National Hospital for Neurology and Neurosurgery, Queen Square, London, WC1N 3BG UK; 4 Sobell Department of Motor Neuroscience & Movement Disorders, UCL Institute of Neurology, Queen Square, London, WC1N 3BG UK

**Keywords:** Benign hereditary chorea, Ataxia, Hypothyroidism, Pituitary gland, Thyroid transcription factor 1

## Abstract

Benign hereditary chorea (BHC) is a rare autosomal dominant condition characterized by early onset, non-progressive chorea, usually caused by mutations in the thyroid transcription factor-1 gene (*TITF1*). We describe a novel mutation arising de novo in a proband presenting in infancy with delayed walking and ataxia. She later developed chorea, then hypothyroidism and a large cystic pituitary mass. Her daughter presented in infancy with delayed walking and ataxia and went on to develop non-progressive chorea and a hormonally inactive cystic pituitary mass. Mutational analysis of the whole coding region of the *TITF1* gene was undertaken and compared with a population study of 160 control subjects. This showed that both affected subjects have a heterozygous A > T substitution at nucleotide 727 of the *TITF1* gene changing lysine to a stop codon at residue 211. Genetic analysis of parents and siblings of the proband confirmed that the mutation arose de novo in the proband. The mutated lysine is an evolutionarily highly conserved amino acid in the protein homoeodomain (HD) where most point mutations associated with BHC are located. The range of mutations in BHC is reviewed with particular emphasis on pituitary abnormalities. Cystic pituitary masses and abnormalities of the *sella turcica* are reported in just 6.4 % of published cases. This is a new nonsense mutation associated with ataxia, benign chorea and pituitary abnormalities which further extends the phenotype of this condition. Mutational screening of *TITF1* is important in cases of sporadic or dominant juvenile-onset ataxia, with mild chorea where no other cause is found, particularly if pituitary abnormalities are seen on imaging.

## Background

Benign hereditary chorea (BHC) (OMIM 118700) is a rare autosomal dominant movement disorder characterized by a non-progressive form of chorea with a prevalence of around two in 10,000,000 [1] and penetrance estimated to be 100 % in men and 75 % in women [2]. Affected individuals may also have pulmonary disease and/or congenital hypothyroidism [3]. For this reason, BHC is described as part of the ‘brain-lung-thyroid syndrome’. Age at onset is usually before the age of 5 years, although this can vary from early infancy to late childhood and adolescence [4, 5]. Delayed motor milestones, specifically late walking, clumsiness and frequent falls, characterize affected children [6]. Speech and intellect are normally unaffected or minimally affected, distinguishing BHC from Huntington’s disease. Progression during adulthood is rare, and there may even be improvement in symptoms. Life expectancy is normal [7]. A general therapeutic treatment is not available, although levodopa administration is sometimes effective particularly for gait difficulties [8]. Other successful treatments have included methylphenidate and corticosteroids [9, 10].

The condition shows considerable intra- and inter-familial phenotypic variability. Additional atypical features include dysarthria and gait disturbances [11], mental impairment [12] or axial dystonia and progression in adulthood [13]. Due to the overlapping symptoms and age at onset, differential diagnosis between BHC and myoclonus-dystonia syndrome is particularly difficult [1, 14, 15]. Both conditions share similar age at onset, dominant inheritance with variable penetrance, minimal progression and the possibility of additional dystonia with no other neurological abnormalities [16].

Breedveld and colleagues [5] first identified a de novo deletion on chromosome 14q13, spanning the *TITF1* gene, which segregated with the disease in a small BHC family. Seventy-seven mutations of the *TITF1* gene have subsequently been identified in BHC patients, including large and small deletions, missense and nonsense mutations. *TITF1* alias *NKX2-1* encodes thyroid-specific transcription factor-1 (TTF-1), a thyroid-specific enhancer-binding protein (T/EBP). The protein was originally identified because it binds to the thyroglobulin promoter and regulates the expression of thyroid-specific genes involved in the production of thyroglobulin, thyroid peroxidase and the thyrotropin receptor. It has also been shown to regulate the expression of genes involved in the secretion of surfactant proteins and Clara cell secretory protein in the lungs [17] and in organogenesis in the thyroid, lung and brain, particularly the ventral forebrain, posterior pituitary and hypothalamus [18–21]. *TITF1* accounts for about 50 % of the mutations associated with BHC suggesting a genetic heterogeneity for this disease [5]. A second BHC locus has been identified in two Japanese families with adult-onset disease which maps to chromosome 8q22.2-q22.3 [22].

## Methods

### Patients

This research was approved by the London (Queen Square) NHS Research Ethics Committee (reference 04/N034) at the National Hospital for Neurology and Neurosurgery, London, UK. The patients were identified through the Ataxia Centre of the National Hospital for Neurology and Neurosurgery. Affected family members provided fully informed signed consent. Patients underwent clinical neurological examination including the scale for the assessment and rating of ataxia (SARA) as well as MR imaging.

### Mutational Analysis

Genomic DNA was amplified by PCR using primer pairs as described by Breedveld and colleagues [5]. The DNA fragments obtained were sequenced by Eurofins MWG Operon Sequencing Service. Paternity was confirmed by using DNA polymorphisms.

### Population Study

A sample of 160 control subjects was screened for the mutation found in our patient through single-strand conformation polymorphism (SSCP) analysis. A 516-bps DNA fragment was amplified by polymerase chain reaction (PCR) with the following primer pair: TITF-3AFN/TITF-3AR [23]. The PCR product was digested with restriction endonuclease BanI. The three fragments obtained (87, 139 and 290 bps) were analysed by SSCP on a GenePhor Electrophoresis Unit (Pharmacia Biotech), using non-denaturing precast acrylamide gels (GeneGelExcel 12.5, Pharmacia Biotech).

## Results

### Proband History

The proband is a 49-year old lady born via spontaneous vaginal delivery after a normal pregnancy. Her mother described her as a ‘floppy’ baby. She began to walk late and with difficulty, holding on to furniture while walking. There were no abnormalities in speech development, but she was diagnosed with cerebellar ataxia at age 2 years. She received intensive physiotherapy and attended a school for disabled children until 16 years old and then completed further education. She has been working and living independently since 18 years of age.

She experienced lifelong balance problems. At around 16 years of age, the balance worsened because of sudden jerky or choreic movements and was started on benztropine at age 17. This improved her symptoms, but she stopped taking this drug at the beginning of her first pregnancy and did not restart it. There have never been any problems relating to swallowing, double vision, bowel or bladder function. She remained well until 40 years of age when she was investigated for weight gain. Hypothyroidism was diagnosed and treated successfully with levothryoxine. The remainder of the pituitary hormonal profile was normal. Respiratory function tests were also normal. Other than this, she was stable between 17 and 40 years of age. Since age 40, she has experienced more jerky involuntary movements and also falls.

On examination, her gait was mildly ataxic, but she was able to walk in tandem and hop on one foot. She had clear choreic jerks and dystonic movements of her head and shoulders and dystonic posturing of the hands while walking. Romberg’s test was negative. Extraocular movements were full, but she had ocular apraxia with difficulty initiating saccades, broken pursuit movements, hypometric saccades and gaze impersistence. The cranial nerve examination was otherwise normal. Mild dysarthria was present. Upper and lower limb tone and power were normal, but there was some dysdiadochokinesia due to intrusion of involuntary movements. There were choreic movements in both feet. The tendon reflexes were present and symmetrical, with flexor plantar reactions and normal sensation throughout. The scale for the assessment and rating of ataxia (SARA) showed gait 2, stance 1, sitting 1, speech 1, finger chase 1–1, nose-finger test 1–1, fast alternating hand movements 1–0, heel-shin slide 1–1.

**Fig. 1:**
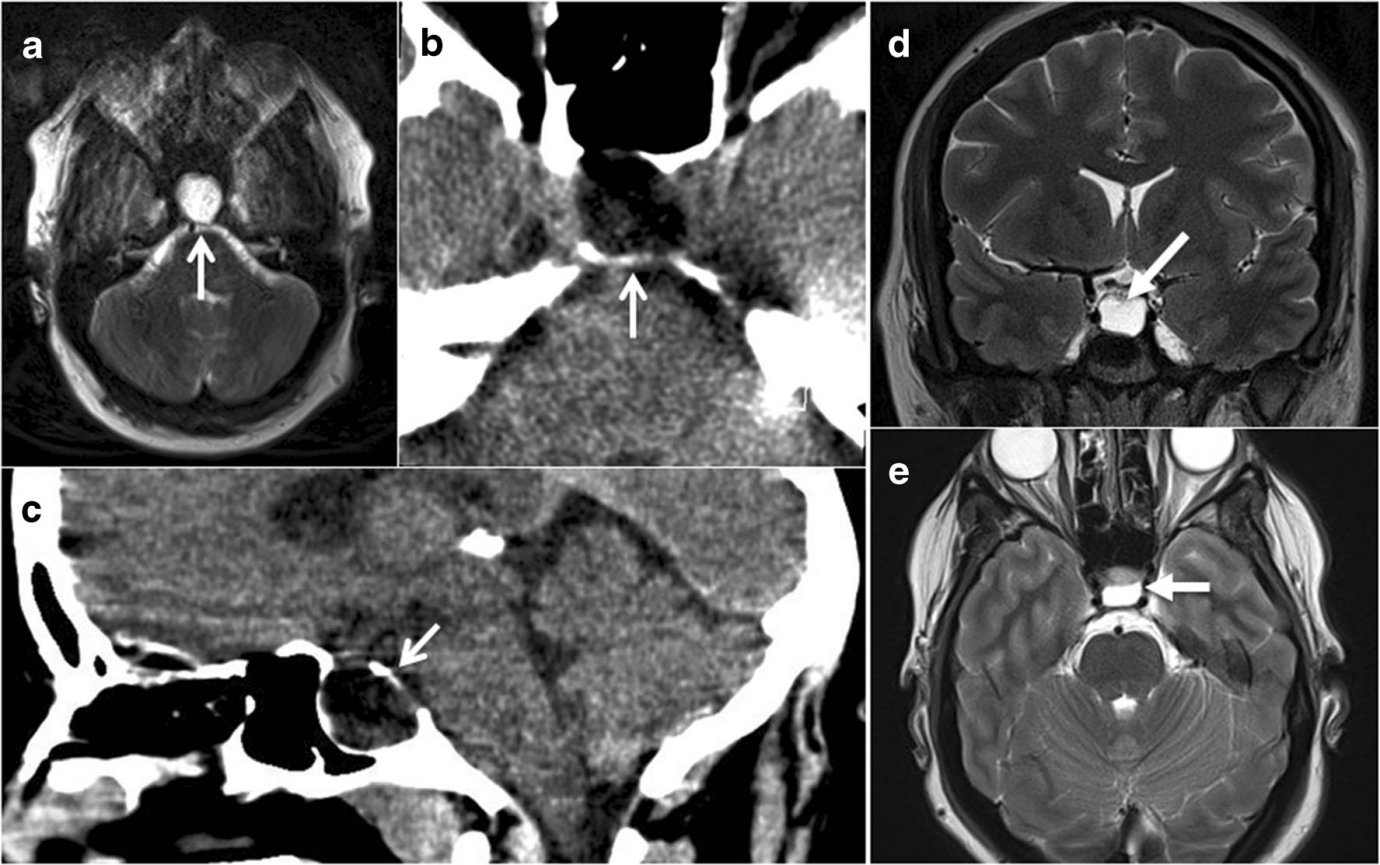
MR imaging. Imaging of proband showing cystic pituitary mass (*open arrow*) **a** T2-weighted axial MRI, **b** axial CT, **c** sagittal CT; imaging of proband’s daughter showing cystic pituitary mass (*solid arrow*) **d** T2-weighted coronal MRI, **e** T2-weighted axial MRI

MR imaging at age 45 showed a cystic structure within an expanded pituitary fossa, abutting but not compromising the anterior visual pathways (see Fig. 1). This was felt to represent an old macroadenoma which had undergone degeneration. The remainder of the brain and brainstem, including the posterior fossa structures, was normal. Subsequent computerized tomography (CT) scan showed a well-defined, rounded, homogeneous lesion of fluid attenuation with erosion and thinning of the sellar walls but without internal calcification, in keeping with a slowly growing cystic lesion.

### Family History

The proband’s daughter is aged 26 and was born via a normal pregnancy and spontaneous vaginal delivery. When she was 3–4 months old, her grandmother noticed she was floppy. She started crawling at 8 months, had problems standing up until well over a year old and did not walk independently until she was 2 years old, due to ataxia. She received intensive physiotherapy that helped her walk more steadily but always had problems maintaining balance on standing. She has had recurrent abnormal jerky movements of all four limbs since childhood, after improvement of her ataxia, which subsequently improved in adulthood. There are no speech problems, but swallowing can be ‘slow’, as she described. She has had recurrent chest infections over many years. There is no formal respiratory diagnosis.

On examination, she had normal gait and tandem walk and was able to hop on one foot. Romberg’s test was negative although there were some choreic movements of the head and legs on standing. There were choreic movements in the upper limbs but no impersistence of the tongue or ‘milking sign’ (alternating contraction and relaxation of the hand). There was no dysarthria. Extraocular movements and speech were unremarkable. Tone, power and coordination were normal. Deep tendon reflexes were present and symmetrical with flexor plantar reflexes and normal sensation. SARA score was 0/40.

MR imaging showed a mildly expanded pituitary fossa with a cyst lying in the posterior aspect of the fossa displacing the infundibulum and adenohypophysis anteriorly (see Fig. 1). The intracranial appearances were otherwise normal with no cerebellar, cerebral or basal ganglia volume loss or abnormal signal change. The only abnormalities on the biochemical pituitary profile were slightly low prolactin level and slightly raised thyroid-stimulating hormone (with normal free T4 level).

**Fig. 2:**
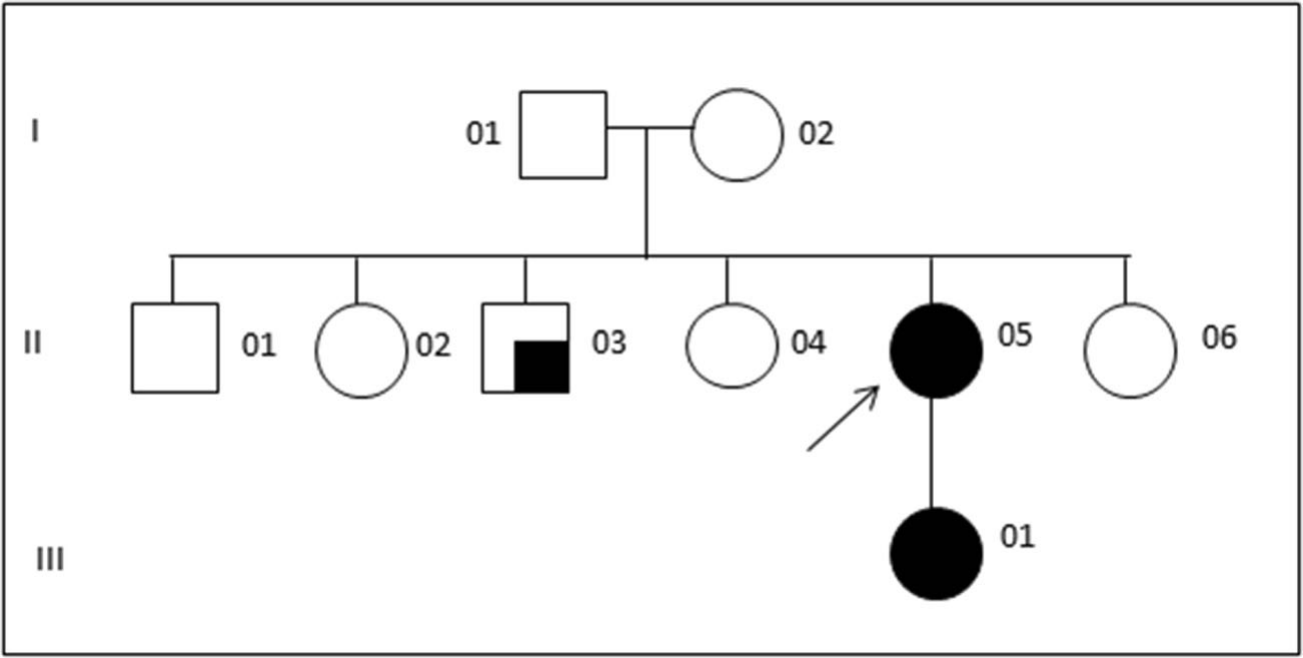
Family tree. Affected individuals II 05 and III 01 carry the K211X mutation. II 03 has clinically confirmed relapsing remitting multiple sclerosis. For I 01, paternity was confirmed via DNA polymorphisms

There is no family history of similar disorders (see Fig. 2). One of the proband’s brothers has clinically confirmed relapsing remitting multiple sclerosis which responds to steroids. He had normal development and no choreic movements. There are four other siblings and seven nieces and nephews, all of whom are unaffected. A paternal aunt had Alzheimer’s disease. The proband’s father had colon cancer. Her mother and siblings were otherwise unaffected. The proband’s mother and one sister were both examined and found to be neurologically normal.

### Genetic Analysis

Sequencing of exons 1, 2 and 3 of the *TITF1* gene of the proband’s DNA revealed a heterozygous A > T variation at nucleotide 727 [GenBank: NM_001079668.2], resulting in a substitution of a lysine residue at codon 211 for a stop codon [K211X, GenePept: NP_001073136.1]. The mutated lysine is an evolutionarily highly conserved amino acid in the protein HD where most of the point mutations associated with BHC are located. The same mutation was identified in her daughter, but not found in her parents, brothers and sisters. The population study excluded the presence of this variation in 160 control European subjects (320 chromosomes).

## Discussion

We describe a novel mutation p.K211X in exon 3 of the *TITF1* gene encoding TTF-1 found in one patient and her child. The mutated protein is predicted to be truncated in the protein HD, located between the amino acids 190 and 249 [24], where the majority of the missense and nonsense mutations associated to BHC are located. This variation is the first de novo nonsense mutation described in *TITF1*, the others being large or small deletions, insertions and missense or splice-site mutations. This mutation segregates in a family with an early onset ataxia followed later with choreic movements.

To date, 78 mutations associated with BHC have been reported in the *TITF1* gene including the present paper (see Table 1). Thirty-four are de novo (43.6 %), 29 (37.2 %) showed an autosomal dominant transmission and, for 15, the transmission was not defined (19.2 %). In one case, the same mutation V235P reported by Krude and colleagues [25] and Uematsu and colleagues [26] is described as a de novo mutation in the first paper and with autosomal dominant transmission in the second. The two patients likely belong to different populations, the first of German origin and the second of Japanese origin. The reason for such a high rate of de novo mutations in this gene is not clear. A reduction of fecundity or fitness conferred by some *TITF1* mutations could be possible. The wide spectrum of symptoms related to *TITF1* mutations may support this hypothesis, including severe respiratory distress leading to an early death [27] and atypical features such as developmental delay [28] and microcephaly [29].

**Table 1:** Mutations in *TITF1* with neurological, thyroid, lung or pituitary involvement

Mutation	Exon	Mutation	Transmission	Pituitary	Brain	Thyroid	Lung	Origin	Reference
Stop	2	p.Y98X	AD	–	+	+	+	Japan	[39]
Stop	2	p.Q107X	AD	–	+	–	–	Spain	[40]
I	2	p.Y116fsX323	De novo	–	+	+	+	NR	[41]
I	2	p.Y116X	AD	NR	+	–	–	NR	[42]
Stop	2	p.C117X	?	NR	+	+	+	NR	[25]
D	2	p.P129fsX307	De novo	NR	+	–	+	US	[43]
Stop	2	p.Y144X	?	NR	+	+	–	France	[44]
Stop	2	p.Y144X	?	NR	+	+	+	US	[43]
SP	2-3	c.463 + 1_463 + 4del	AD	NR	+	+	–	NR	[42]
SP	2-3	c.463 + 1G > A	De novo	–	+	+	+	Spain	[45]
SP	2-3	c.464-9C > A	AD	–	+	+	–	Japan	[46]
SP	2-3	c.464-1G > A	De novo	–	+	+	+	Spain	[47]
SP	2-3	c.464-2A > C	AD	–	+	–	–	UK	[1]
SP	2-3	c.464-2A > T	AD	–	+	–	NR	Canada	[31]
SP	2-3	c.464-2A > G	AD	–	+	+	+	US	[48]
SP	2-3	c.464-2A > G	De novo	–	+	+	+	France	[49]
D	3	p.S163fsX2	De novo	NR	+	+	+	NR	[42]
Stop	3	p.S175X	AD	Empty sella	+	+	+	Italy	[28, 34]
D	3	p.P185fsX250	De novo	NR	+	+	+	US	[43]
I/D	3	p.P187fsX196	De novo	–	+	+	+	Japan	[50]
D	3	p.R195fsX32	AD	NR	+	+	+	Brazil	[51]
MS	3	p.R195W	De novo	NR	+	+	+	US	[43]
MS	3	p.L197P	?	NR	+	–	+	US	[43]
MS	3	p.F198L	AD	NR	–	–	+	US	[43]
MS	3	p.F198L	?	NR	–	–	+	US	[43]
MS	3	p.F198L	?	NR	–	–	+	US	[43]
Stop	3	p.S199X	?	NR	+	+	–	NR	[25]
Stop	3	p.E205X	AD	–	+	–	–	Germany	[8]
MS	3	p.L206V	De novo	–	+	–	–	France	[42, 49]
Stop	3	p.R208X	AD	–	+	–	–	UK	[23]
Stop	3	p.K211X	De novo	Cystic mass	+	+	–	UK	Present study
MS	3	p.Y215D	De novo	NR	+	+	–	NR	[42]
Stop	3	p.S217X	AD	–	+	+	–	Ashkenazi Jewish	[52]
MS	3	p.L224R	AD	NR	+	+	–	NR	[42]
I	3	p.A225fsX228	De novo	NR	+	–	–	NR	[25]
MS	3	p.P233L	De novo	NR	+	+	–	France	[49]
MS	3	p.V235P	De novo	Cystic mass	+	+	+	NR	[25]
MS	3	p.V235P	AD	–	+	+	–	Japan	[26]
MS	3	p.I237F	De novo	NR	NR	+	+	French Canadian	[27]
MS	3	p.I237M	De novo	NR	–	+	+	US	[53]
MS	3	p.W238L	AD	–	+	NR	NR	US	[5]
MS	3	p.Q240P	De novo	–	+	+	–	France	[42, 49]
MS	3	p.R243S	AD	–	+	NR	NR	Netherlands	[5]
MS	3	p.R243P	AD	NR	+	–	–	NR	[42]
Stop	3	p.Y244X	AD	NR	+	+	–	NR	[42]
Stop	3	p.Q249X	AD	–	+	–	–	Portugal	[54]
D	3	p.G266del	AD	–	+	NR	+	Italy	[55]
I	3	p.G269_271dupGGG^a^	?	NR	+	–	+	US	[43]
I/D	3	p.274_280del7aa and p.G273fsX152	?	NR	+	+	+	US	[43]
D		p.A280fsX161	AD	NR	+	+	+	France	[44]
D	3	p.L293del	De novo	NR	+	+	+	NR	[42]
D	3	p.G303fsX77	AD	–	+	NR	NR	UK	[5]
D	3	p.A306fsX350	AD	–	+	+	–	Spain	[56]
D	3	p.Q327fsX121	De novo	–	+	+	+	Netherlands	[3]
I	3	p.H349fsX90	De novo	NR	+	+	+	US	[43]
D	3	p.Q357fsX24	AD	–	+	–	–	NR	[57]
D	3	p.S366fsX67	?	NR	–	+	+	US	[43]
I	3	p.T389fsX52	?	NR	+	+	+	US	[43]
LD		del 14q13-q21	De novo	–	NR	+	+	Belgium	[58]
LD		del 14q12-q13.3	De novo	–^b^	+	+	+	NR	[29]
LD		del 14 1.2 MB	De novo	–	+	NR	NR	Italy	[5]
LD		del 14 1.2 MB	AD	Stalk duplication	+	+	NR	Italy	[35]
LD		del 14q11.2-q13.3	?	Cystic mass	+	+	+	NR	[25]
LD		del 14q13	De novo	–	+	+	+	France	[49]
LD		del 14 0.9 MB	AD	–	+	+	+	France	[59]
LD		del 14q12-q13	De novo	–	+	+	+	Japan	[26]
LD		del 14q13.2-q22.1	De novo	NR	+	–	–	NR	[42]
LD		del 14q13.2-q21.2	De novo	NR	+	+	–	NR	[42]
LD		del 14q13.3	De novo	NR	+	+	–	NR	[42]
LD		del 14q13.1-q21.1	De novo	NR	+	+	+	US	[43]
LD		del 14q13.3	?	NR	+	+	+	US	[43]
LD		del 14q13.3-q21.1	De novo	NR	+	+	+	US	[43]
LD		del 14q13.1-q21.1	De novo	NR	+	+	+	US	[43]
LD		DEL ex1-2	?	NR	+	+	+	US	[43]
LD		del 14q13.2-q21.1	De novo	–	+	+	–	Australia	[60]
LD		del 14q13.3	AD	NR	+	+	+	France	[44]
LD		del 14q13.3	De novo	NR	+	+	–	France	[44]

*SP* splicing mutation, *I/D* small insertion/deletion, *LD* large deletion, *M* missense, *AD* autosomal dominant, *NR* not recorded, *?* unknown

^a^Two unrelated patients

^b^No pituitary abnormality but cystic mass of cavum septum pellucidum

The high rate of de novo mutations and incomplete penetrance has significant implications for clinical diagnosis and genetic counselling. Since evidence of autosomal dominant inheritance is absent in many cases of BHC, mutational screening of *TITF1* is important not only in cases of juvenile-onset chorea but also in early onset ataxia where no other cause is found, as was the case with our case when she first presented. Importantly, the risk of transmission to subsequent generations remains 50 % even in such seemingly sporadic cases. Mutational screening should also be considered in cases of autosomal dominant early onset ataxia with extracerebellar signs—the so-called ADCA type I according to the Harding classification—if other more common causes have been excluded and particularly if chorea is present [30].

The cause of the proband’s ataxia is not clear as there was no evidence of cerebellar or frontal atrophy on imaging and no clinical evidence of sensory neuropathy. Histopathological studies in BHC are few and associated with sparse changes. Kleiner-Fisman et al. [31] found no gross or microscopic abnormalities apart from some mild frontal-parietal-temporal atrophy and non-specific astrocytosis of the globus pallidus, thalamus, hippocampus and periaqueductal grey matter. Subsequent re-examination of the same brain showed a reduced density of striatal met-enkephalin- and substance-P-immunoreactive nerve fibres [32]. Of note, this patient did not show cerebellar ataxia. Therefore, in our case, we hypothesize that there may be a dysfunction of the basal ganglia-cerebellar loops [33] responsible for the cerebellar syndrome.

The distribution of point mutations in the *TITF1* gene causing BHC is uneven. No mutations are described in exon 1, whereas the acceptor splice site at exon 3 appears to be a mutational hot-spot with six mutations affecting this site reported. Mutations truncating the protein span the whole gene affecting all protein domains. Missense mutations associated with BHC are exclusively located in the HD and not in other protein regulatory domains, presumably impairing the binding activity of the transcription factor with DNA.

Cystic pituitary masses and abnormalities of the *sella turcica* are reported in just a few cases: five out of the 78 cases hitherto published (6.4 %) (see Table 1) associated with missense and nonsense mutations and large deletions [25, 28, 34, 35]. Silberschmidt and colleagues [36] defined in vivo the role of two different *TITF1* transcriptional activation domains flanking the nuclear localization signal (NLS) and the HD, namely the N-terminal (N-AD) and the C-terminal (C-AD) activation domains. These portions of the protein have different developmental functions. In particular, the N-AD seems to be essential for pituitary morphogenesis whereas both are essential for thyroid morphogenesis. None of the reported mutations associated with pituitary defects is located in the N-AD. However, this is not necessarily surprising, since truncated proteins are unable to translocate into the nucleus, notwithstanding the presence of the NLS in many cases [28], probably impairing the function of N-AD. Furthermore, the remarkable phenotypic heterogeneity observed in BHC families showing pituitary abnormalities suggests that other factors may modify *TITF1* activity in pituitary development. These may include other genes, transcription factor haploinsufficiency of various causes [37] and epigenetic mechanisms [38]. Much further work remains to elucidate this problem.

## Abbreviations

BHC: Benign hereditary chorea
C-AD: C-terminal activation domain
CT: Computerized tomography
HD: Homeodomain
MR: Magnetic resonance
N-AD: N-terminal activation domain
NKX2-1: NK2 homeobox 1 gene
NLS: Nuclear localization signal
PCR: Polymerase chain reaction
RET: Receptor tyrosine kinase gene
SARA: Scale for the assessment and rating of ataxia
SSCP: Single-strand conformation polymorphism
T/EBP: Thyroid-specific enhancer-binding protein
TITF1: Thyroid transcription factor-1 gene
TTF-1: Thyroid-specific transcription factor-1
